# Supplementary material for: Breathing-driven prefrontal oscillations regulate maintenance of conditioned-fear evoked freezing independently of initiation
Source: Nat Commun. 2021 May 10;12:2605. doi: 10.1038/s41467-021-22798-6 (PMC8110519; doi:10.1038/s41467-021-22798-6)
Supplement: Supplementary file 1 — Supplementary Information [file 41467_2021_22798_MOESM1_ESM.pdf]

Supplementary Materials

Supplementary discussion

Evidence from spinal cord lesion studies

As discussed in the main text, clear evidence in humans that the subjective experience of emotions is impaired with diminished bodily feedback has been hard to come by. Most studies have focused on spinal cord lesion patients and results have been overall inconclusive. Our findings point out two possible explanations for this and lead to some experimentally testable predictions. First, in spinal cord lesion patients, feedback of the breathing rhythm via the olfactory system will still be functioning, as well as other pathways such as the vagus nerve. Second, most studies are directed at measuring the intensity of emotions and not their temporal evolution. Our results with optogenetic perturbation of the OB and therefore the respiratory feedback show that this specifically impacted the maintenance but not the initiation of emotional states.

We therefore tentatively predict that impaired bodily feedback could lead to discontinuous or shorter emotional experiences. For example, what appears to be a sustained emotional state could in fact be supported by multiple initiations of short emotional bouts. Studies will need to be designed to specifically test this possibility.

The temporal evolution of emotions linked to bodily feedback has never been directly addressed. Going back to the literature on spinal cord lesion patients there are however some striking remarks that suggest that emotional maintenance has been affected (Hohmann, 1966):
"Now I get kinda mad one minute, and **the next minute it'll be like nothing happened**."
"Where I used to blow my stack, feel all shaky and that sort of thing, and I would strike out and get in several fights, now many less things upset me, **and the upset has left me before I know it**."
"Well, I believe I feel a little less tension when I get mad now. I don't shake so much, and it is easier to control. **Maybe I get over it a little quicker**, but I always did get over it fairly quickly anyway."

G.W. Hohmann, Psychophysiology (1966) 143–156.

Calculation of model probabilities

The expected number of steps in a freezing bout duration (E(FzDur)), can be derived from the probability that the bout will last n steps :

$$P(FzDur = n) = {P_{Fz/Fz}}^{n} \times (1\text{-}P_{Fz/Fz})$$

$$E(FzDur) = \sum_{n=0}^{+\infty} n \times {P_{Fz-Fz}}^{n} \times \left(1 - P_{Fz/Fz}\right)$$

$$E(FzDur) = (1 - P_{Fz-Fz}) \times P_{Fz-Fz} \times \frac{d}{dP_{Fz-Fz}} \sum_{n=0}^{+\infty} {P_{Fz/Fz}}^{n}$$

$$E(FzDur) = (1 - P_{Fz-Fz}) \times P_{Fz-Fz} \times \frac{d}{dP_{Fz/Fz}}\left(\frac{1}{1-P_{Fz/Fz}}\right) \quad \text{as} \quad \sum_{i=0}^{n} q^{i} = \frac{1-q^{n+1}}{1-q}$$

$$E(FzDur) = \frac{P_{Fz/Fz}}{1 - P_{Fz/Fz}}$$

The expected number of steps in an active bout duration can be derived in the same fashion.

$$E(ActDur) = \frac{P_{Act/Act}}{1 - P_{Act/Act}}$$

It should be noted that the function x/(1-x) is monotonically increasing for x>0, so that we can directly interpret an increase in the measured $E(FzDur)$ as an increase in $P_{Fz/Fz}$ and, respectively, $E(ActDur)$ as an increase in $P_{Act/Act}$.

Fig.S1 Electrode localisation

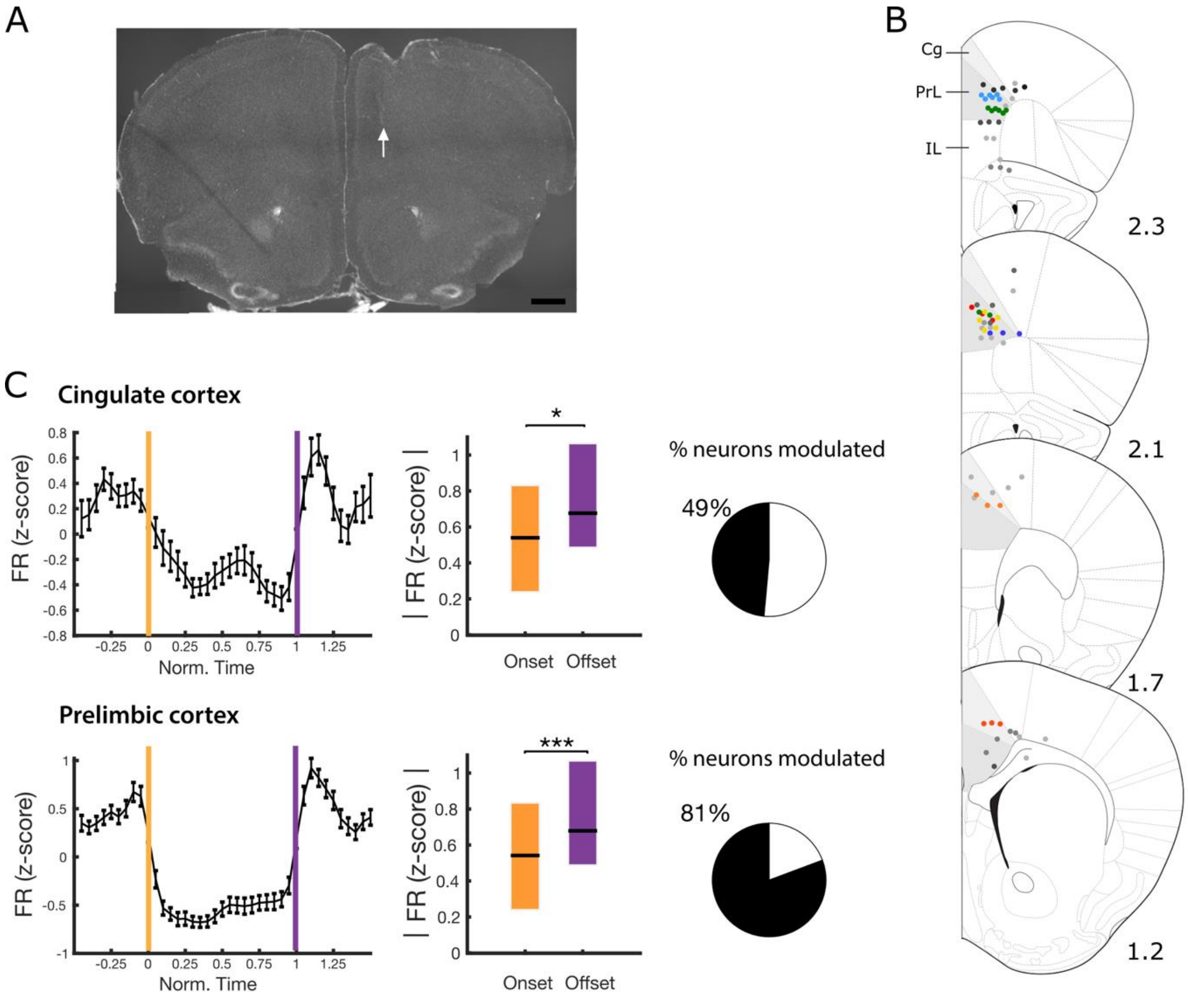

A. The recording sites were reconstructed post hoc from coronal brain sections as shown in this example. Example taken from n=18 histologies. The white arrow indicates the electrode track. Black bar: 500µm

B. Recording locations for 18 mice (5 /18 mice were implanted bilaterally) with antero-posterior distance from Bregma (mm) on the right for each section. Electrodes were localized to target areas in dorsomedial prefrontal cortex (shaded in grey). Colored dots represent recordings sites of animals in which 100% tetrodes were in prelimbic and cingulate cortex only and which were used for neural analysis whereas grey dots show excluded locations. Dots are color coded by animal. Schematic line drawings were adapted from the Paxinos and Watson (1998) rat brain atlas.

C. Neural analysis presented in Figure 5 is performed on neurons located both in the cingulate and prelimbic cortes. In a subset of animals tetrodes were exclusively located in one of the two cortices, allowing for separate analysis (cingular cortex on top, n= 69 and prelimbic cortex below, n= 57). Both groups of neurons show the same dynamics as the whole neuron population but the effects are clearer for prelimbic neurons.

Left. Z-scored response of units during normalized freezing episode. Error bars are SEM. Note that for all units, the response has been sign-corrected (units that increase firing during freezing are multiplied by -1) to allow averaging of responses that displays the amplitude of their freezing modulation, independent of the sign.

Middle. Median response (absolute value) of units at onset (yellow) and offset (purple) of freezing. Boxplots show median and interquartile range. Wilcoxon signed rank test, Cingular cortex : zval=–2.53, p=0.0116, n=69, Prelimbic cortex : zval=-4.27, p=$1.93.10^{-5}$, n=57).

Right. Percent of significantly modulated units by OB 4Hz (black).

Fig. S2. Bulbectomy related changes in behaviour

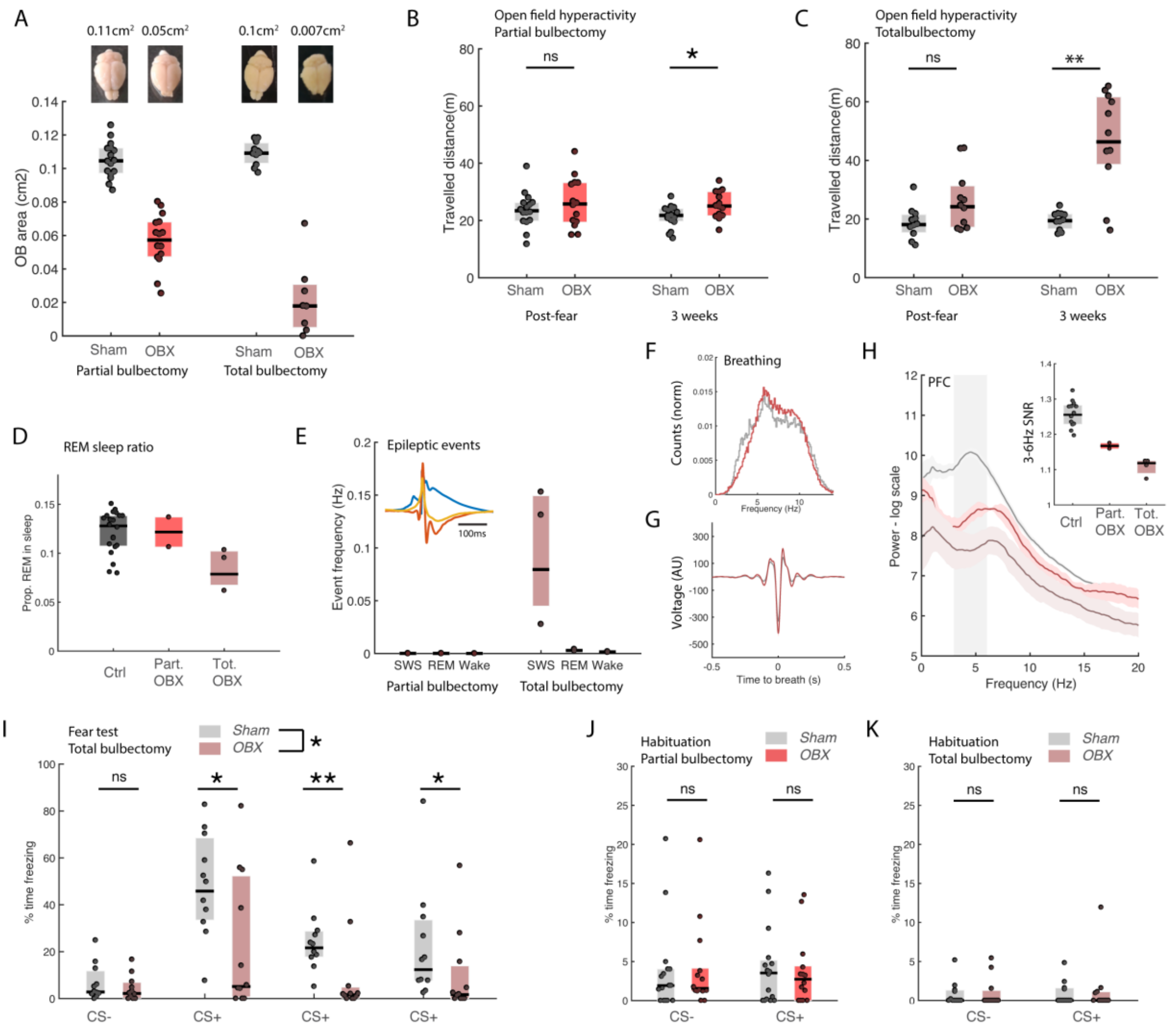

A. Area of OB after surgery for sham and bulbectomized mice using either a technique of partial or total OB ablation, measured using photographs such as those shown above of representative lesions from each group (n= 16, 15, 11, 8 respectively). Note the clear separation in extent of remaining OB area which justifies dealing with experiments separately. In the following, we document the overall behavioural changes for these two kinds of bulbectomy.

B. Travelled distance in the open field test after 10min for sham and partially bulbectomized (OBX) mice, at two time points: the day following the fear test and three weeks after bulbectomy. Hyperactivity only appears three weeks after surgery. (Wilcoxon rank sum test, zval = 0.64, 2,34; p=0.519,0.018; n=16 & 14 mice)

C. As in B for totally bulbectomized mice that show a weak but non-significant tendency for hyperactivity at the time of the experiment and a very pronounced hyperactivity after three weeks. (Wilcoxon rank sum test, zval=1.773, 2.955 p=0.076 0.003, n=11 & 11 mice, n=11 & 11 mice). D. Proportion of sleep spent in Rapid Eye Movement (REM) sleep for a group of control mice (n = 20), partially bulbectomized mice (n=2) and totally bulbectomized mice (n=3). Note that although total bulbectomy leads to a reduction in REM sleep, partial bulbectomy has no apparent effect.

E. Frequency of large events reminiscent of inter-ictic spikes suggestive of epilepsy as measured in the HPC in partially bulbectomized mice (n=8, no events detected) and totally bulbectomized mice (n=15, high frequency of events during NREM sleep). Note that during wake neither group showed any signs of epilepsy. Inset shows the event-triggered average of the detected spikes from the three totally bulbectomized mice that were recorded.

F.G. Sham and fully bulbectomized mice were placed in the plethysmograph to measure any changes in

breathing. We found the distribution of breathing frequency (F) and the overall shape of each breath (G) was very similar between both groups, indicating a lack of change in respiratory pattern.
H. Averaged power spectra during freezing in sham (blue, n=15) and partially (n=8) or totally bulbectomized (n=11) (red) mice, as shown grouped together in Fig1D. Error bars are SEM. Inset: signal-to-noise ratio of 3-6Hz band during freezing periods. Note that both partial and total bulbectomy lead to a drastic loss of 4Hz power during freezing.
I. Median freezing levels of sham and totally bulbectomized mice during the test session. Note the similar reduction in overall freezing levels as seen for partial bulbectomy in Fig3A. (Two-way mixed repeated measures anova: group ($p=0.041$, $F=4.73$) x CS block ($p<0.0001$, $F=16.28$), interaction ($p=0.09$, $F=2.18$). Post-hoc Wilcoxon ranksum on each individual block: zval=0.82, 2.1, 2.6296, 2.2389; p=0.41, 0.05, 0.01, 0.02, n=11,11; Effect size for significant differences: 0.95,0.79, 0.56)
J.K Median freezing levels of sham and totally bulbectomized mice during the pre-conditioning habituation session. No differences were found between the two groups, reinforcing the finding in the open field that no overall motor change confounds our results. (Partial OBX : Wilcoxon rank sum test, ranksum value : 250, 259, p=0.95, 0.65, n=16 & 14 mice; Total OBX : Wilcoxon rank sum test, ranksum value : 133, 128, p=0.64, 0.90, n=11 & 11 mice).
In all panels, boxplots show median and interquartile range.

Fig. S3. dmPFC-OB coherence is preserved when using local bipolar signal

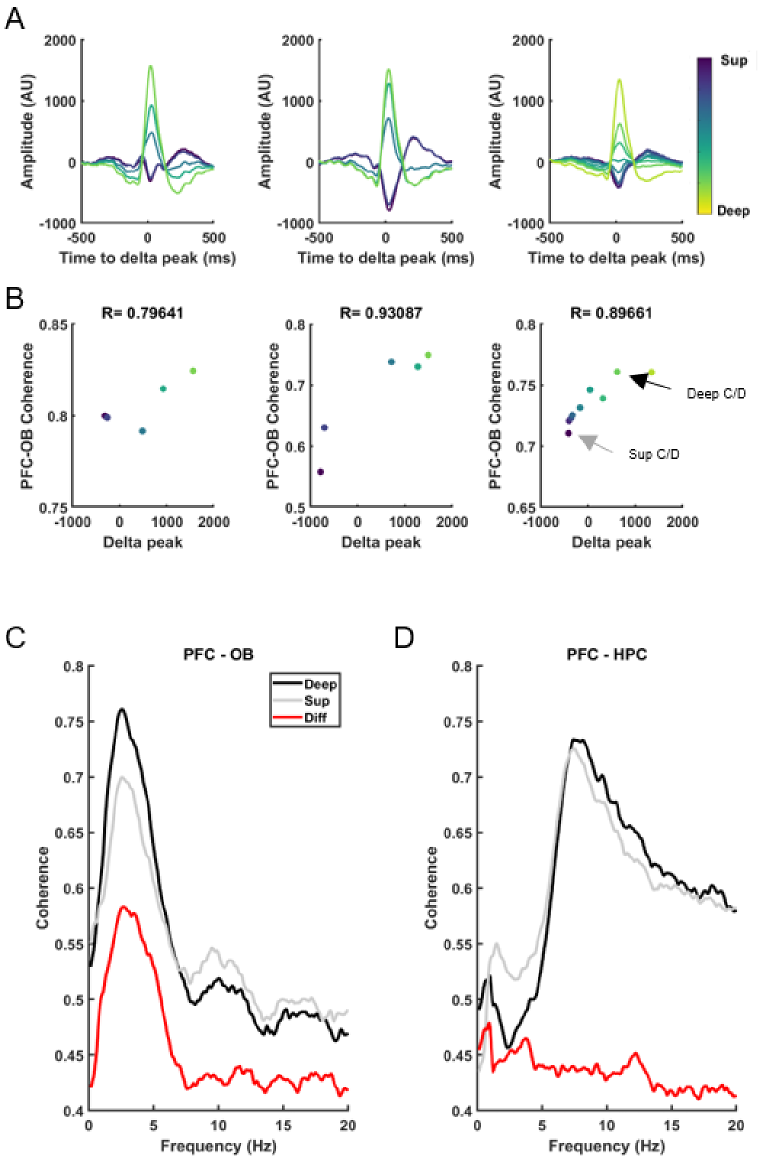

A. LFP triggered on delta waves from electrodes at multiple depths in the dmPFC. The amplitude and sign of the response is a good indicator of electrode depth, which allowed us to assess the depth of dmPFC electrodes. Data from 3 mice with broad spread of recording depths. Inferred depth is color coded from light green (deep) to dark blue (sup)

B. Correlation of OB-dmPFC coherence during freezing for each dmPFC electrode and the amplitude of delta peak used as a proxy for recording depth. For each mouse there is a very strong correlation (measured using Spearman correlation, $p<0.01$), showing that the 4Hz in the dmPFC is related to depth.

C. For the two dmPFC electrodes shown by arrows in B, coherence with the OB shows the lower coherence in the superficial layer. A pseudo-local signal can be generated by subtracting the LFP from the two electrodes (Diff, red) and shows clear coherence with OB. This strongly suggests that at least some component of the 4Hz signal is not volume conducted from the OB.

D. Same as in C but for dmPFC coherence with HPC. Note that deep and superficial layer electrodes show very similar levels of coherence in the theta band and the pseudo-local signal generated by subtraction completely abolishes coherence, suggesting that the HPC theta is volume conducted

Fig. S4. Transmission of optogenetically induced oscillations in OB to dmPFC cannot be explained by volume conduction

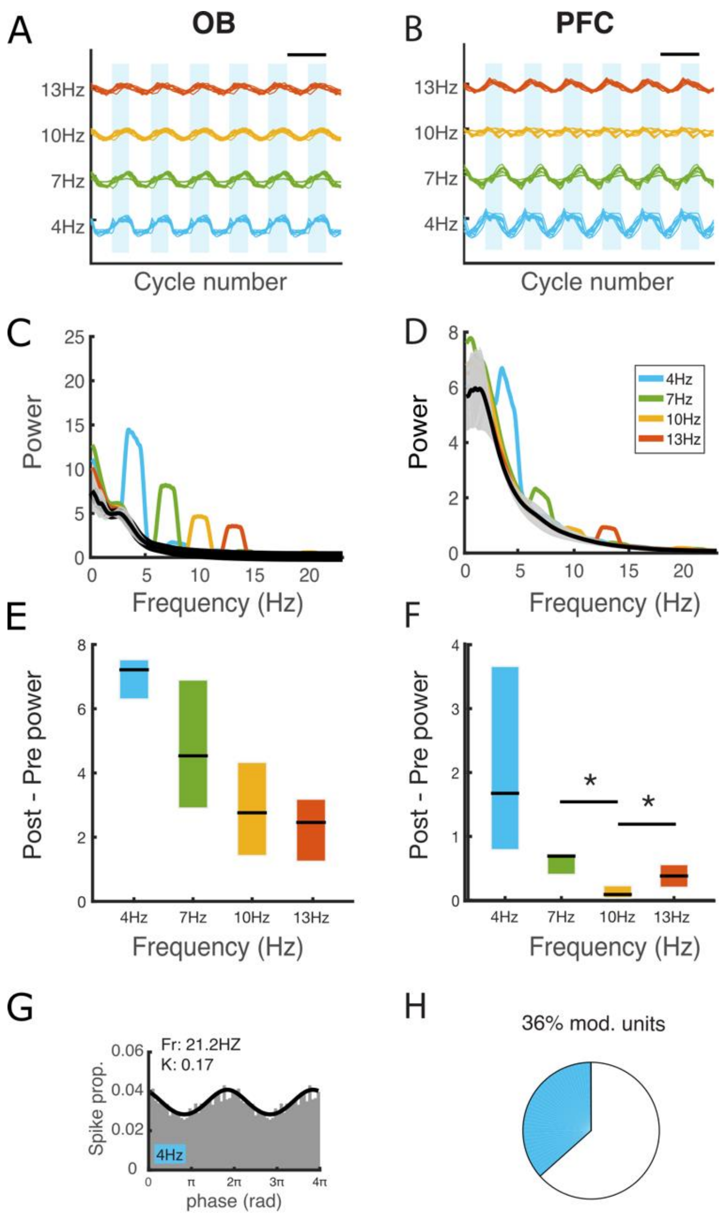

A. B. LFP triggered on laser stimulation showing a constant number of cycles for OB and dmPFC. The OB shows clear oscillations at each frequency. On the contrary, only the 4Hz, 7Hz and 13Hz oscillations in the OB evoke dmPFC modulation, whereas at 10Hz almost no entrainment is visible. The passive electrical properties of biological tissues cannot produce the non-linear transfer function between OB and dmPFC unveiled by our optogenetic stimulation of the OB. For each mouse, all responses are concatenated and z-scored. (7 recordings sessions from 4 mice). Black bar : 250ms.

C. D. Average spectra in OB and dmPFC before ChR2 stimulation in the OB (gray) and during stimulation (colored). To average between mice with varying implantation depths, the spectra from each animal are normalized to total power. (n=4 mice)

E. F. Median difference in power between the pre-stimulation and stimulation period at each frequency for OB (E) and dmPFC (F). * : 10Hz is lower than neighbouring frequencies (Friedman test p = 0.045 in both cases, n=4). Boxplots show median and interquartile range.

G. Phase histogram of an example dmPFC unit modulation by OB during 4Hz stimulation.
H. Percentages of dmPFC units modulated by the OB LFP during 4Hz stimulation using Rayleigh's test with p=0.05., (n=100 units).

Fig. S5. dmPFC single unit recordings and entrainment by respiratory related rhythm

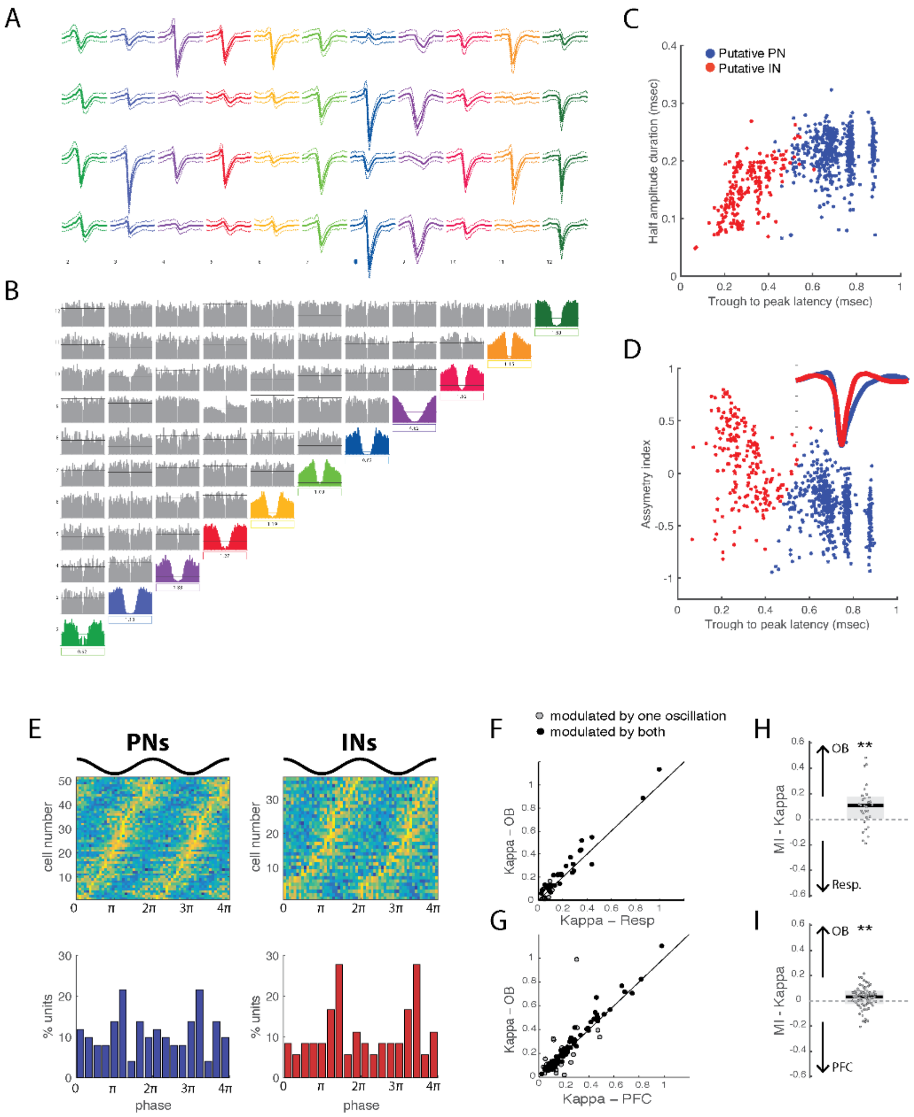

A. B. Example spike sorting sessions showing mean waveforms on each wire of the tetrode and cross and auto-correlograms for each identified single unit.

C. D Waveforms were clustered using three waveform characteristics: duration at half-amplitude, trough to peak latency and the asymmetry index (positive ratio of the difference between right and left baseline-to-peak amplitudes and their sum) Putative pyramidal neurons (blue) and interneurons (red) were clustered using k- means algorithm. Inset shows mean waveforms of all pyramidal neurons (blue) and interneurons (red).

E. Phase distribution of all significantly modulated dmPFC PNs and INs by OB LFP (top) and distribution of preferred phases for all significantly modulated PNs and INs (bottom).

F. Correlation of the von Mises concentration coefficient (K) of dmPFC units during freezing relative to respiration and the 4Hz oscillation in the OB LFP. Significantly modulated units for either or both signals are shown.

G. As for F but for 4Hz oscillation in the dmPFC vs the OB LFP.

H. Modulation index of the von Mises concentration coefficient (K) showing significantly stronger modulation for the OB signal than the respiration signal for units that are modulated by both signals. (Wilcoxon rank sum test, $p=0.0032$, zval=2.95, $n=30$ units).

I. As for H but for 4Hz oscillation in the dmPFC vs the OB LFP. (Wilcoxon rank sum test, $p=0.0029$, zval=2.98, $n=76$ units).

H-I: boxplots show median and interquartile range.

Fig. S6. Reduced dmPFC locking to respiratory rhythm after methimazole injection

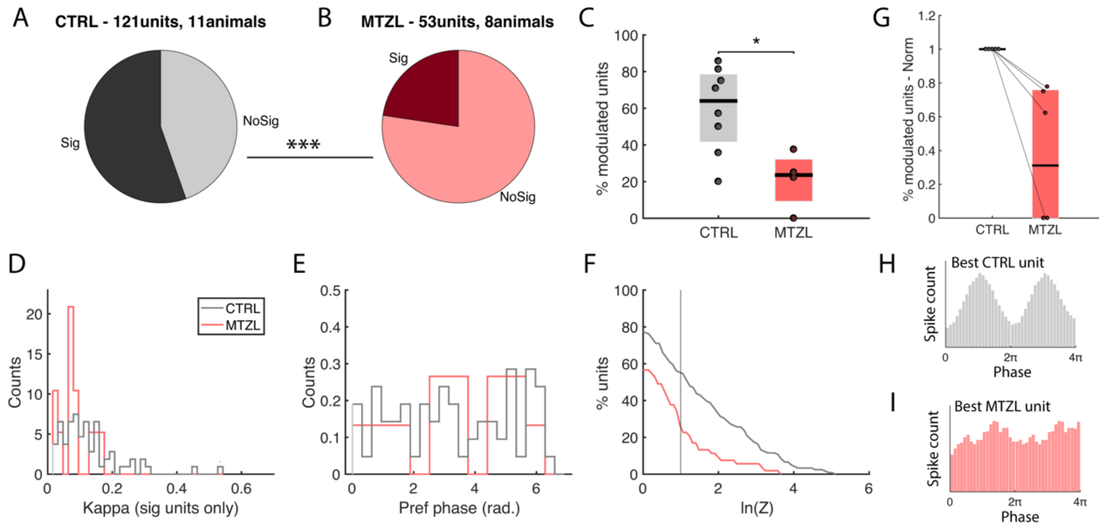

A. B. Overall proportion of units significantly modulated by breathing in control and methimazole-injected animals during the post-conditioning test session. Chi2 test shows that more units are modulated in control animals (chi2stat=14.63, p=1.3E-4, n=121,53 units)
C. Proportion of units significantly modulated by breathing in each control and methimazole-injected animal with more than 6 simultaneously recorded neurons. injection (Wilcoxon ranksum test, ranksum statistic: 64, pval= 0.0485, n= 8&4).
D. Distribution of concentration coefficient Kappa for all significantly modulated units. Note the weaker modulation depth of units from methimazole injected animals.
E. Distribution of preferred phase for all significantly modulated units.
F. Cumulative distribution of log-transformed Rayleigh's test Z of dmPFC units modulation by breathing.
G. A subset of animals (n=5) were recorded both before and after methimazole injection and showed systematic decrease in number of modulated units.
H. I. Unit with highest kappa recorded in saline and methimazole animals respectively
In all panels, boxplots show median and interquartile range.

Fig. S7. Increased response of dmPFC to OB input during freezing relative to active periods

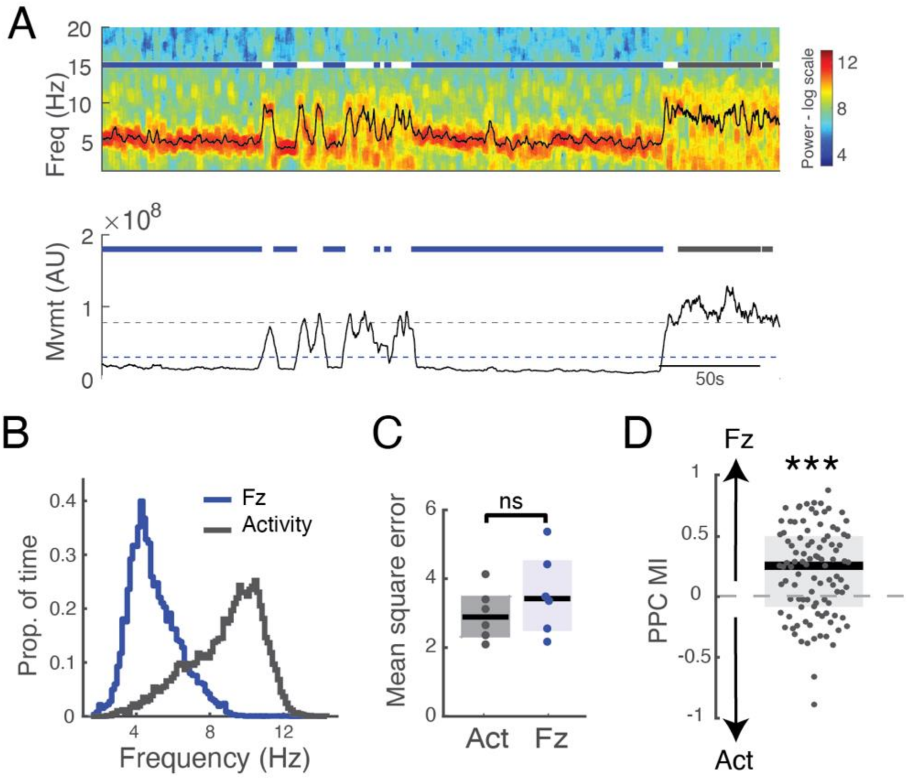

A. Example spectrogram of OB activity showing the instantaneous frequency estimated using the peak-to-peak phase estimation method (black line). Note that the instantaneous frequency closely tracks the strongest band of the spectrogram despite changes in frequency. Below the quantity of movement illustrates the definition of freezing (below the blue line) and active (above the red line) epochs. The active threshold was adjusted to have equivalent total duration of data for both epochs. Red and blue lines represent active and freezing epochs respectively.

B. Distribution of instantaneous frequencies of OB LFP during freezing and active periods. Freezing frequencies peak around 4Hz whereas active frequencies show a broader distribution around 10Hz. Note that these frequencies are slightly higher the breathing frequencies shown in Fig1C because in the restricted volume of the pletysmograph mice could not attain high speeds and here only time points of active exploration are used, not merely all time points outside of freezing which may include quiet wakefulness.

C. Mean square error between the LFP signal in the OB and the reconstructed signal using interpolated phase estimated using the peak-to-peak method. Error rates were not significantly different during the active and freezing periods, indicating that this method allows us to track the phase of the despite changing frequencies. (Paired Wilcoxon signed rank test: signed rank statistic: 5; p=0.31, n=6 mice).

D. Modulation index of the pairwise phase consistency index for dmPFC units relative to OB LFP showing a significant increase in spiking phase modulation during freezing relative to active epochs for the OB signal only. This measure is robust to changes in firing rate. Arrows on the left indicate how the figure can be interpreted: positive values indicate an increase in phase modulation during freezing behaviour. (One-sample two-sided Wilcoxon signed rank test with 0: zval= 5.06, p=4.2e-07, n=100 units).

In all panels, boxplots show median and interquartile range.

Fig. S8. Firing rate controls

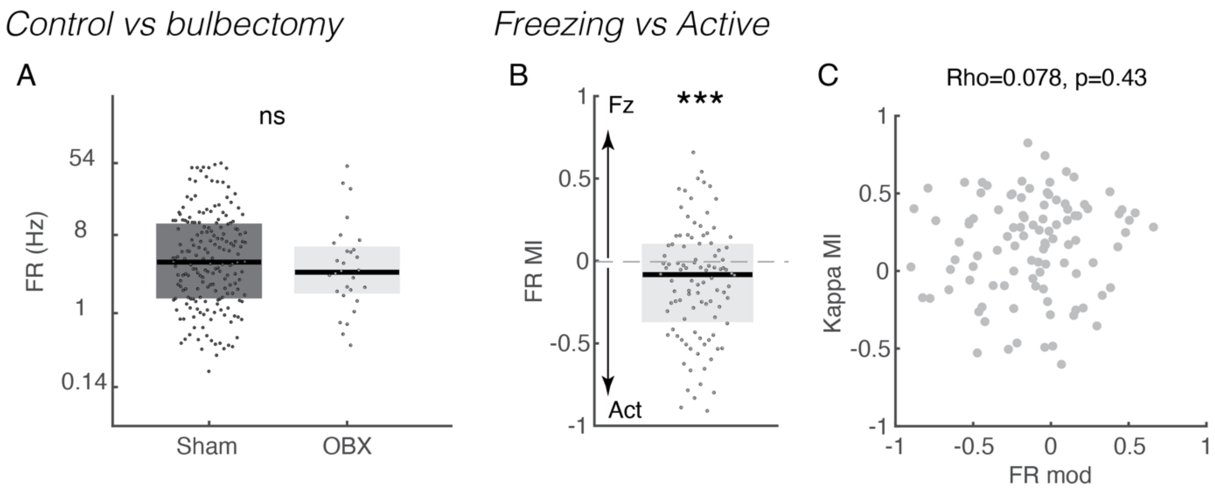

A. Firing rates of dmPFC units in control and bulbectomized mice, showing no difference in overall level of activity. (Wilcoxon rank sum test, zval=0.8986, p=0.3689, n=191 units in 13 control mice, 29 units in 3 bulbectomized mice).

B. Modulation index of firing rate of all neurons between freezing and active periods showing that on average units decrease their firing rate during freezing. (One-sample two-sided Wilcoxon signed rank test with 0: zval=-3.4314, p=6.0e-04, n=100 units)

C. Modulation index of concentration coefficient Kappa as a function of the modulation index of firing rate of all neurons showing the absence of correlation. (Spearman correlation coefficient: R=0.078, p=0.43, n=100 units).

In all panels, boxplots show median and interquartile range.

FigS9. Specificity of optogenetic stimulation

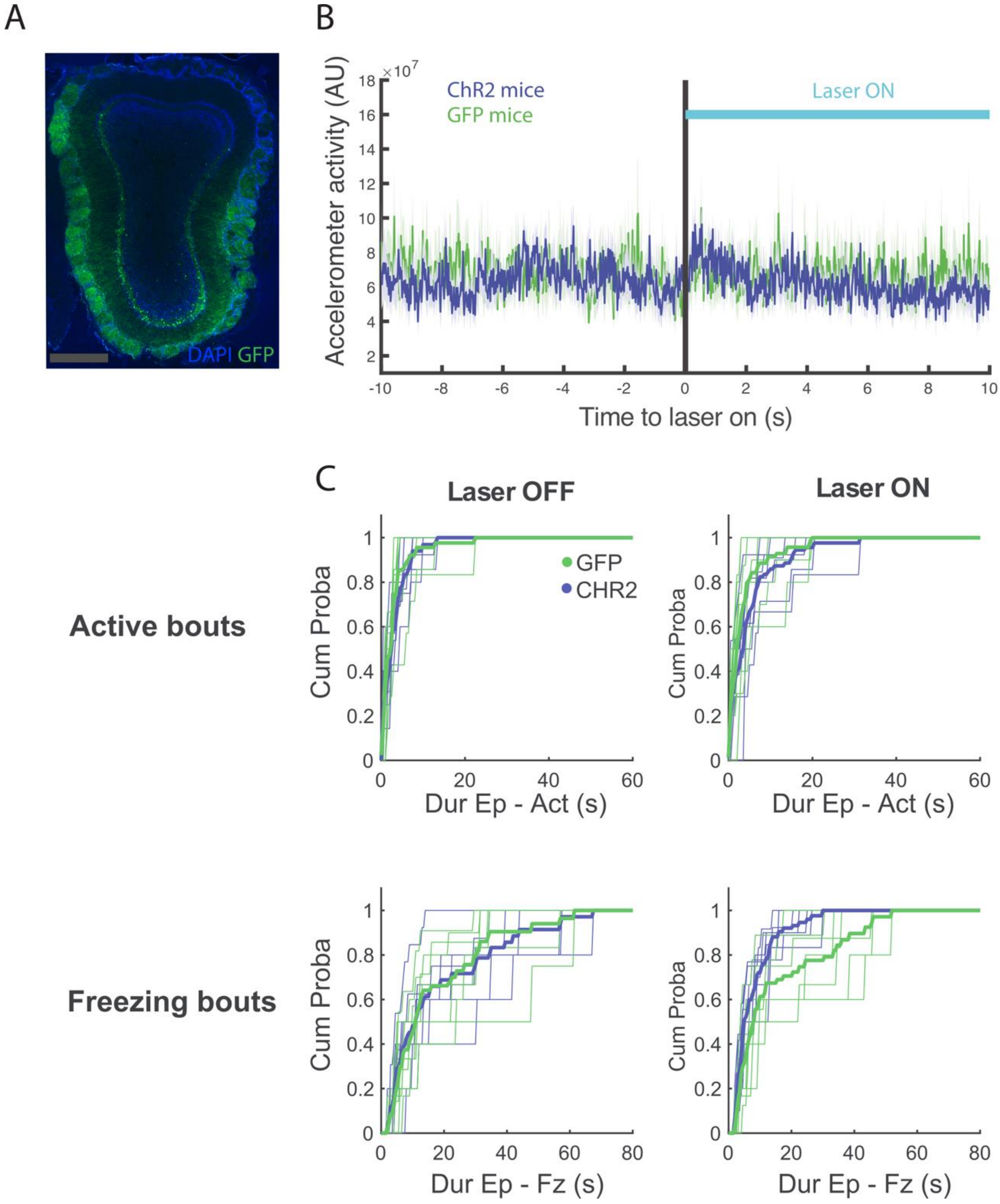

A. OB histology of ChR2 injected mice. Note the presence of ChR2+ cells in the glomerular layer. Scale bar = 400 µm.

B. Movement of GFP and ChR2 infected mice before and after 13Hz laser stimulation during the habituation session. Stimulation does not induce any changes if overall behaviour. Error bars are SEM (n=7&7).

C. Cumulative distribution of active and freezing bout lengths for GFP and ChR2 expressing mice during CS+ presentations before (left) and during (right) laser 13Hz stimulation. Note that without stimulation the distributions are nearly identical. During stimulation, although active bout duration curves remain similar, there is an upwards shift of the freezing bout duration curve for ChR2-expressing mice relative to their GFP controls, indicating a reduction in freezing bout duration.

Fig. S10. Wide-ranging behavioural and physiological changes induced by methimazole injection

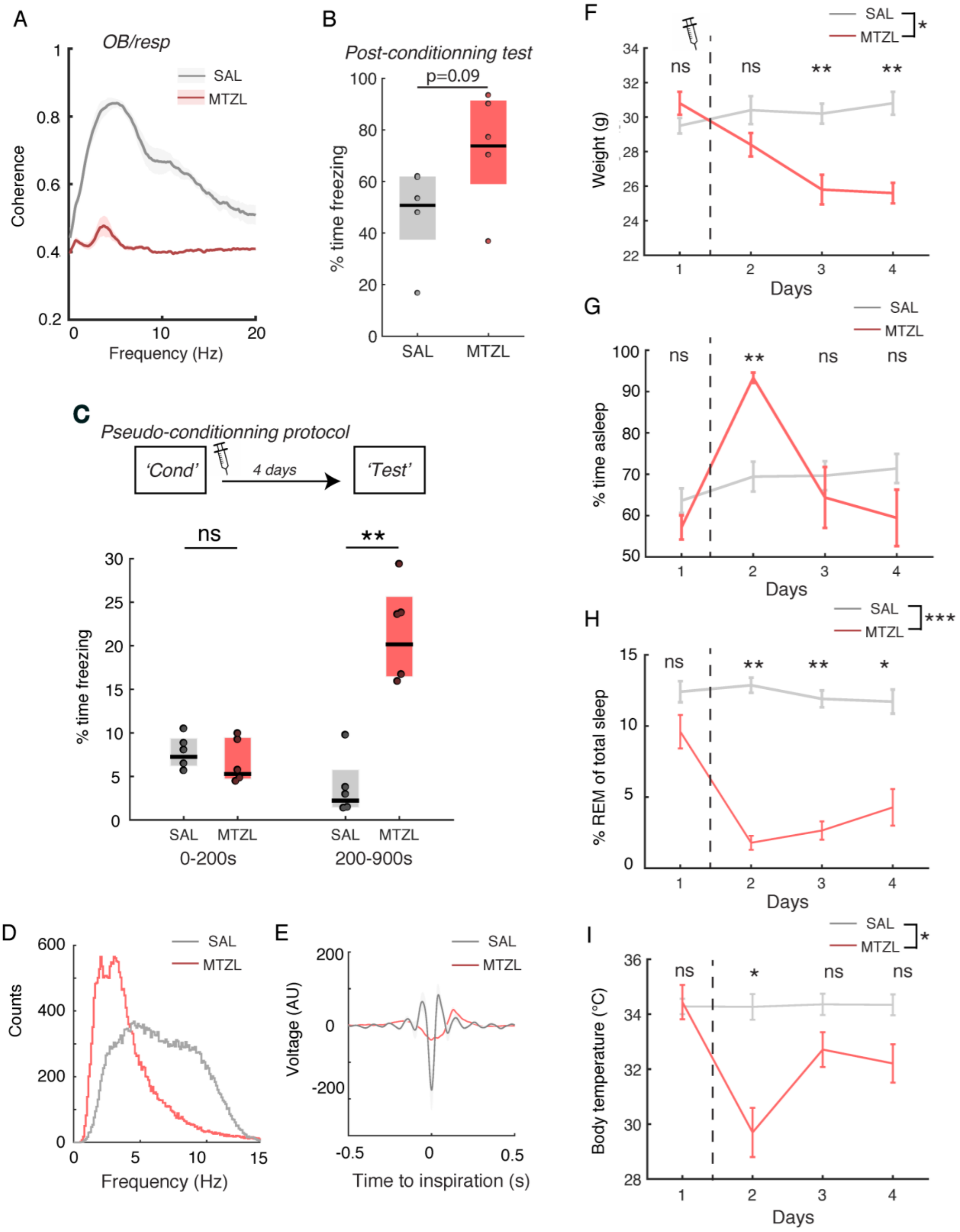

A. Averaged coherence between breathing and the olfactory bulb during whole test session in saline (gray) and methimazole (red) injected mice. Error bars are SEM. (n=5 & 5 mice)
B. Freezing levels of saline and methimazole injected mice during CS+ presentation in the test session after our tone conditioning. Note the trend to increased levels in methimazole mice, similar to what was observed in [24], despite the differences in conditioning and testing protocols. (Wilcoxon signed rank test: Signed Rank statistic=19, p=0.09; n=5 & 5 mice).
C. Freezing levels of saline and methimazole injected mice during the test session after a pseudo-conditioning protocol. This protocol reproduced the conditionning protocol used in Moberly et al by placing mice in two environments for 15min however no shocks or sounds were delivered. This allowed us to observe the non-specific effects of methimazole injection on behaviour. Note that after 200s, methimazole injected mice show an increase in time spent freezing despite there being no prior conditionning and no stimulus presentation. The time periods used correspond exactly to those used in[24] which were pre- and post-tone. We replicate closely their observations during these two periods suggesting that these results can be attributed to non-specific, broad changes in behaviour induced by methimazole injection. (Wilcoxon signed rank test: Signed Rank statistic=15, 31, p=0.547, 0.0079, n=5,5).
B-C. Boxplots show median and interquartile range.
D. Distribution of breathing frequencies for all mice during total post-conditioning test session. Methimazole injected mice breathe much slower than the saline control injected animals.
E. Inspiration-triggered average of breathing trace showing the typical shape of breathing. Note the strong change in overall shape and the slowing of the breath in methimazole mice, in particular contrasting with the absence of change in bulbectomized animals (FigS1F,G).
F. Evolution of weight showing strong reduction in methimazole injected compared to saline injected mice. Error bars are SEM. (Two-way mixed repeated measures anova: group (p=0.0185, F=8.69) x day (p<0.0001, F=31.01), interaction (p=<0.0001, F=45.11). Post-hoc Wilcoxon ranksum on each individual block, ranksum statistic: 14.5,35,40,40; pval: 0.27, 0.13, 0.0079, 0.0079, n=5 & 5 mice).
G. Overall time spent sleeping after saline or methimazole injection. Note that there appears to be a strong increase of sleep only on the day after injection (Wilcoxon ranksum test, ranksum statistic: 15, pval= 0.0025). This non-linear effect of time may explain the non-significant effects of group in anova test. Error bars are SEM. (Two-way mixed repeated measures anova: group (p=0.9937, F=0.00) x day (p<0.0013, F=6.7), interaction (p=<0.0043, F=5.39); n=5 & 7 mice)
H. REM sleep loss after saline or methimazole injection (Two-way mixed repeated measures anova: group (p<0.0001, F=86.87) x day (p<0.0045, F=5.36), interaction (p=0.0042, F=5.42). Post-hoc Wilcoxon ranksum on each individual bloc: ranksum statistic: 41,50,50,45, pval: 0.2,0.0025,0.0025,0.048, n=5 & 7 mice).
I. Reduced body temperature after methimazole injection compared to saline (Two-way mixed repeated measures anova: group (p=0.043, F=5.74) x day (p<0.0001, F=11.7), interaction (p=0.0001, F=10.91). Post-hoc Wilcoxon ranksum on each individual bloc: ranksum statistic: 28,39,35,31 pval: 1, 0.015, 0.15, 0.19, n=5 & 5 mice).
F-I : Error bars are SEM.

Fig. S11. Optogenetic stimulation of the OB at 4Hz

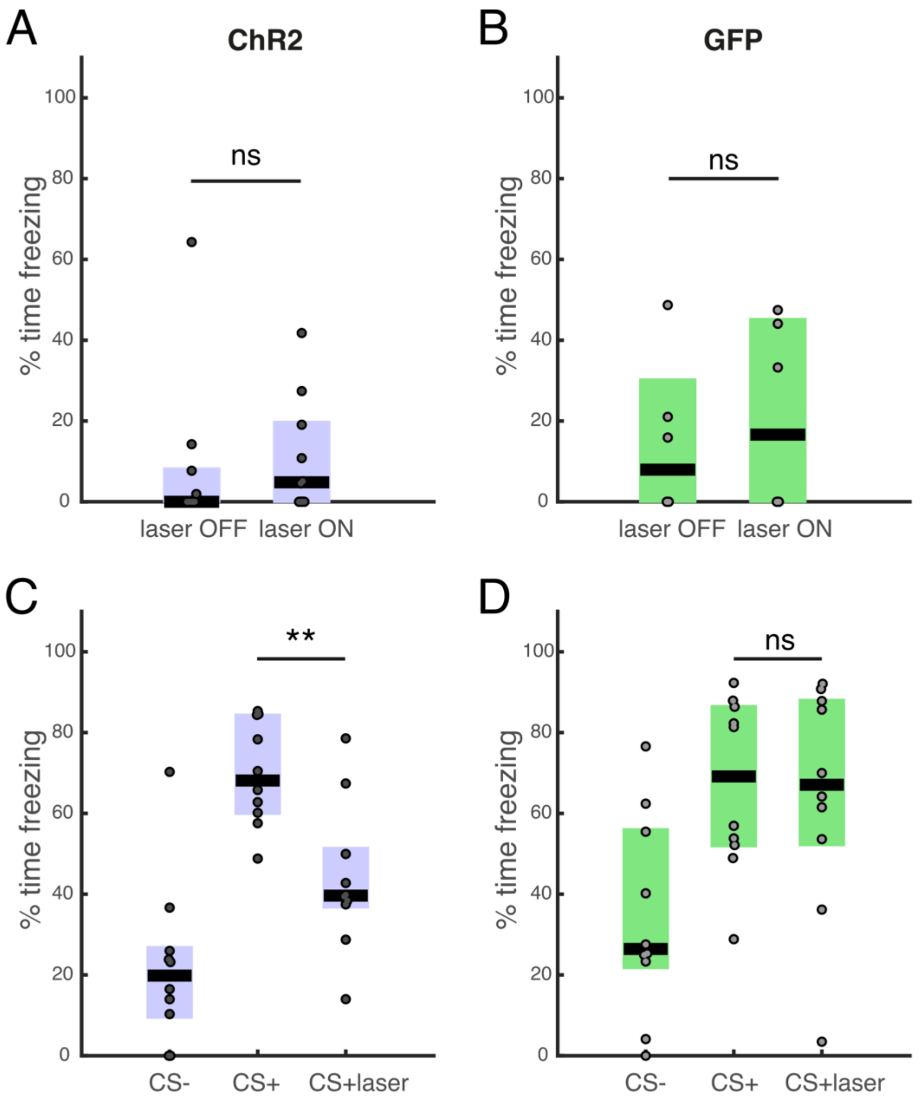

A,B. 4Hz-stimulation to unconditioned mice did not affect the freezing level, that remained low for both ChR2 (n=10) and GFP mice(n=5). These results are consistent with the hypothesis of a role for 4Hz in the maintenance of already initiated freezing episodes : since freezing events are unlikely in unconditioned animals we do not expect any effect of the stimulation. (Wilcoxon signed rank test: Signed Rank statistic=94, 26, p=0.396, 0.825, n=10,5)
C,D. 4Hz optogenetic stimulation during CS+ in conditioned animals reduces freezing behaviour in ChR2(n=10) mice and not the GFP controls(n=10). This result is very similar to the effect of 13Hz stimulation and may result from the same physiological effect: scrambling of OB output and perturbation of its effect in the dmPFC. Indeed, when the optogenetic 4Hz stimulation is applied, the OB is also oscillating at around 4Hz. External and endogenous oscillations will randomly occur in or out of phase. Therefore 4Hz stimulation does not enhance the 4Hz oscillation but will instead tend to perturb the regular, oscillatory character of the OB output. Only a closed-loop system where stimulation is triggered at a given phase of the oscillation would allow to assess the sufficiency of 4Hz for freezing. (Wilcoxon signed rank test: Signed Rank statistic=142, 103, p=0.006, 0.910, n=10,10; Effect size for ChR2 with/without stimulation: 1.65; Effect size ChR2 vs GFP during stimulation: 0.88).
In all panels, boxplots show median and interquartile range.

Fig. S12. Markov model of freezing behaviour

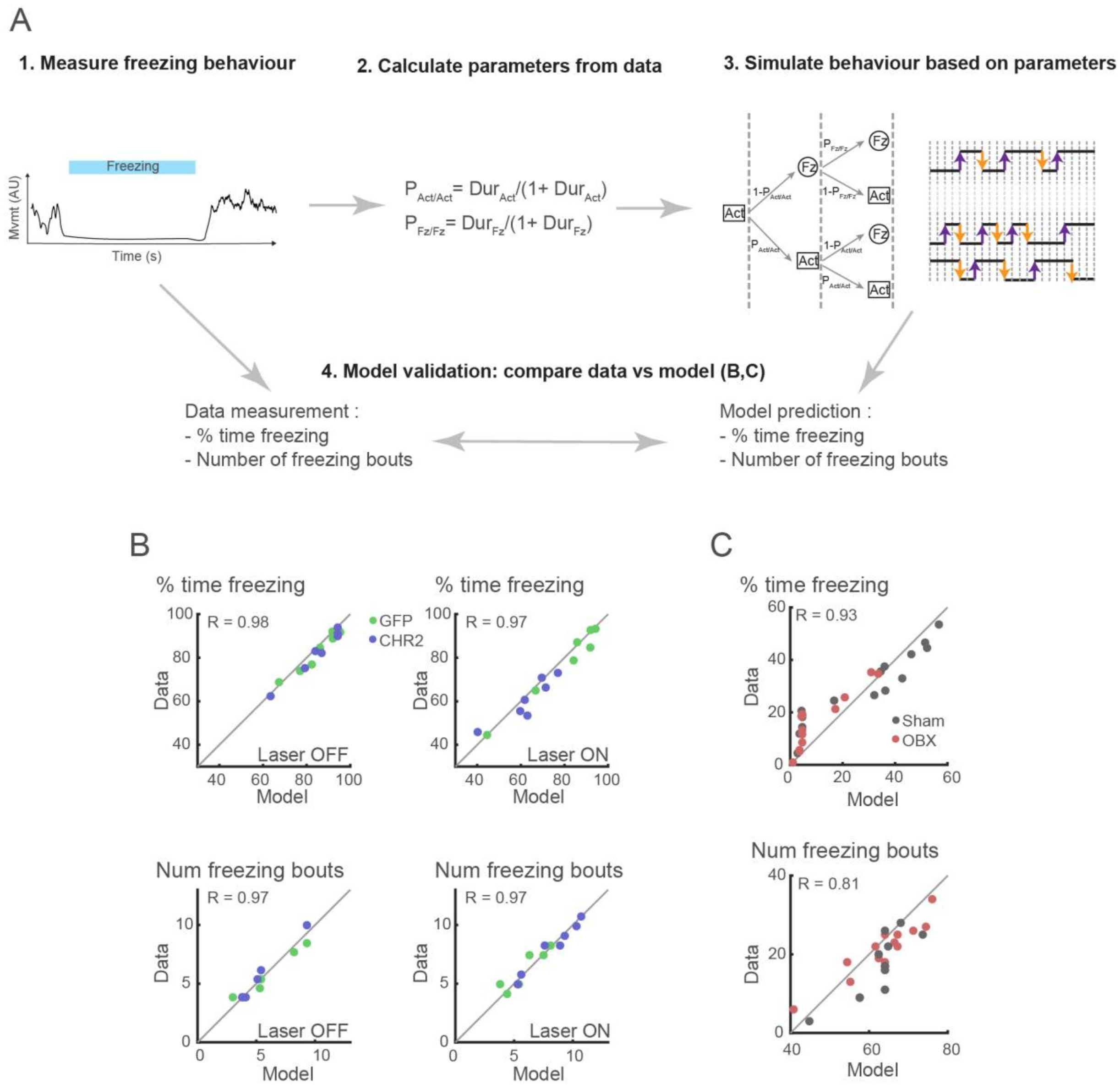

A. Flowchart showing the establishment and validation of the Markov model used to model freezing. Based on freezing data (step 1), the two probabilities ($P_{Act/Act}$ and $P_{Fz/Fz}$) can be directly calculated from the average active and freezing bout durations (step 2, see supplementary discussion for demonstration). Based on these two probabilities, time courses of freezing activity can be stimulated : at each time step, depending on the current state (Fz or Act), the next state is randomly selected according to these probabilities (step 3). Finally, to establish the quality of the model, two other parameters not used to calibrate the model, the percent of time spent freezing and the number of freezing bouts, are estimated from these simulations and compared to data (step 4).

B.C. Correlation of data with simulation-based prediction of the Markov model for the total percent of time spent freezing and the number of freezing bouts for GFP and ChR2 animals (B) and sham and bulbectomized animals (C). Note that in all cases, there is a high correlation coefficient (all p values < 1E-8) and points lie close to the identity line shown in black, indicating an excellent prediction by the model.

Fig. S13. 4Hz power and dmPFC single units prediction of freezing state at freezing onset and offset

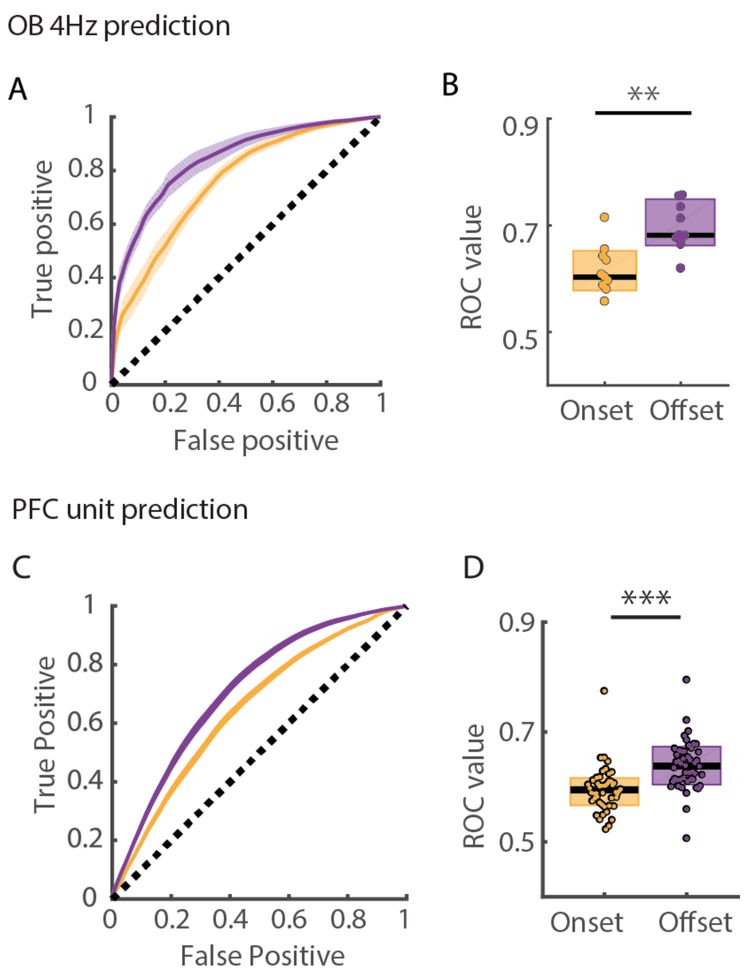

A. Receiver operating curves using power in the 4Hz band to predict freezing or non-freezing state either at the onset (blue) or offset (red) of freezing. The black line indicates the curve expected by chance. Error bars are SEM.

B. Area under the ROC curve freezing onset and offset. (Wilcoxon signed rank test: Signed Rank statistic=55, p=0.0019, n=10 mice)

C. D As A and B, for dmPFC single units activity. (Wilcoxon signed rank test: zval=4.83, p=1.35e-6, n=48 single units)

In all panels, boxplots show median and interquartile range.

Fig. S14. Correlation between the duration of a freezing epoch and the 4Hz power

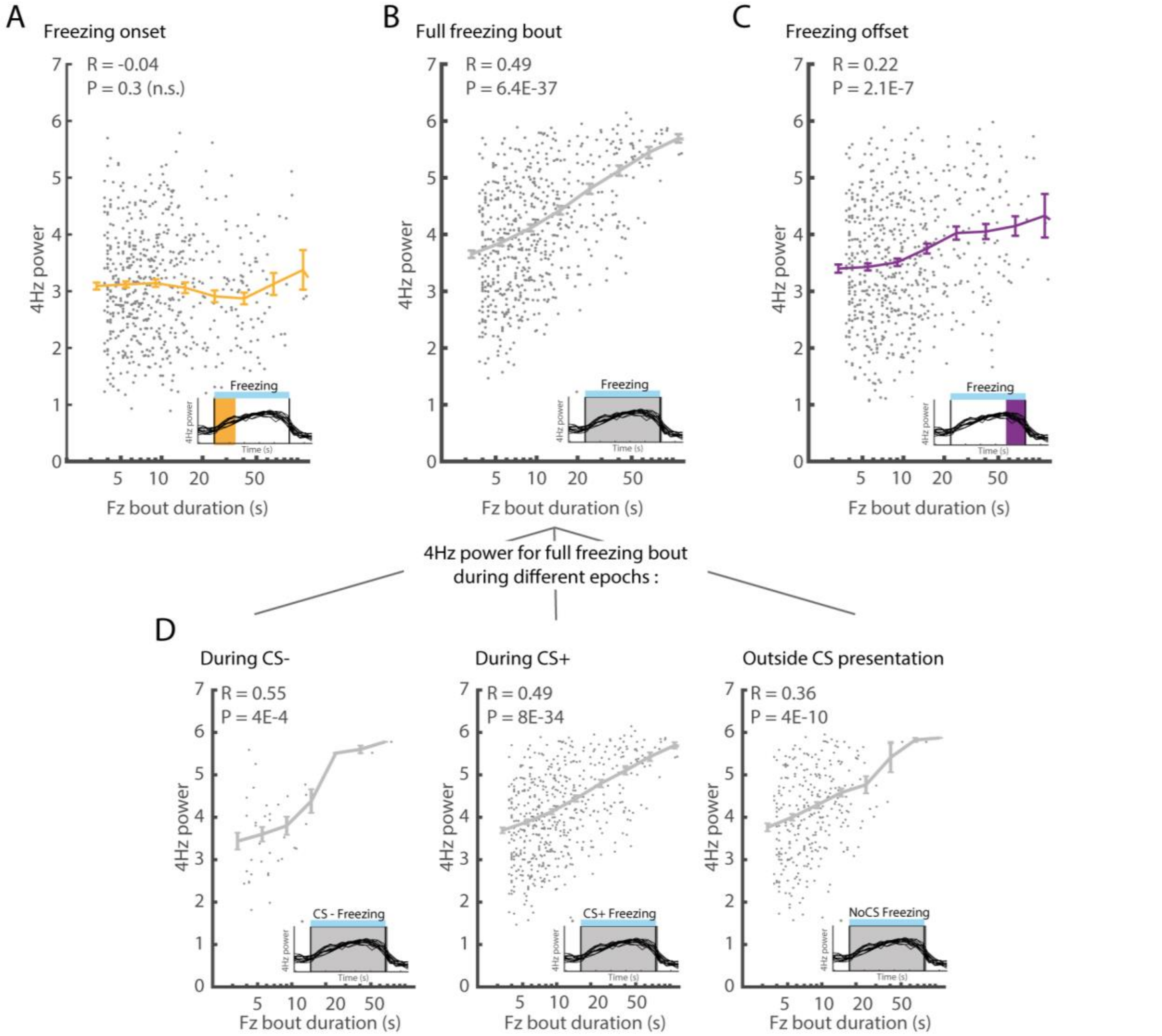

A. B. C. Pearson correlation between the duration of a freezing bout and the 4Hz power (A) at onset (during the first 2s), (B) during the full bout and (C) just before offset (during the last 2s). (n=11 mice). Error bars are SEM. Insets indicate the epochs used in reference to the evolution of 4Hz power during freezing.

D. Pearson correlation between the duration of a freezing bout and the 4Hz power during the full bout as in B but split into three different epochs of the test session: CS-, CS+, absence of CS. For all epochs, the correlation between 4Hz and freezing duration is highly significant. (n=11 mice). Error bars are SEM.

Fig. S15. Methodology to study evolution of population activity throughout freezing episode

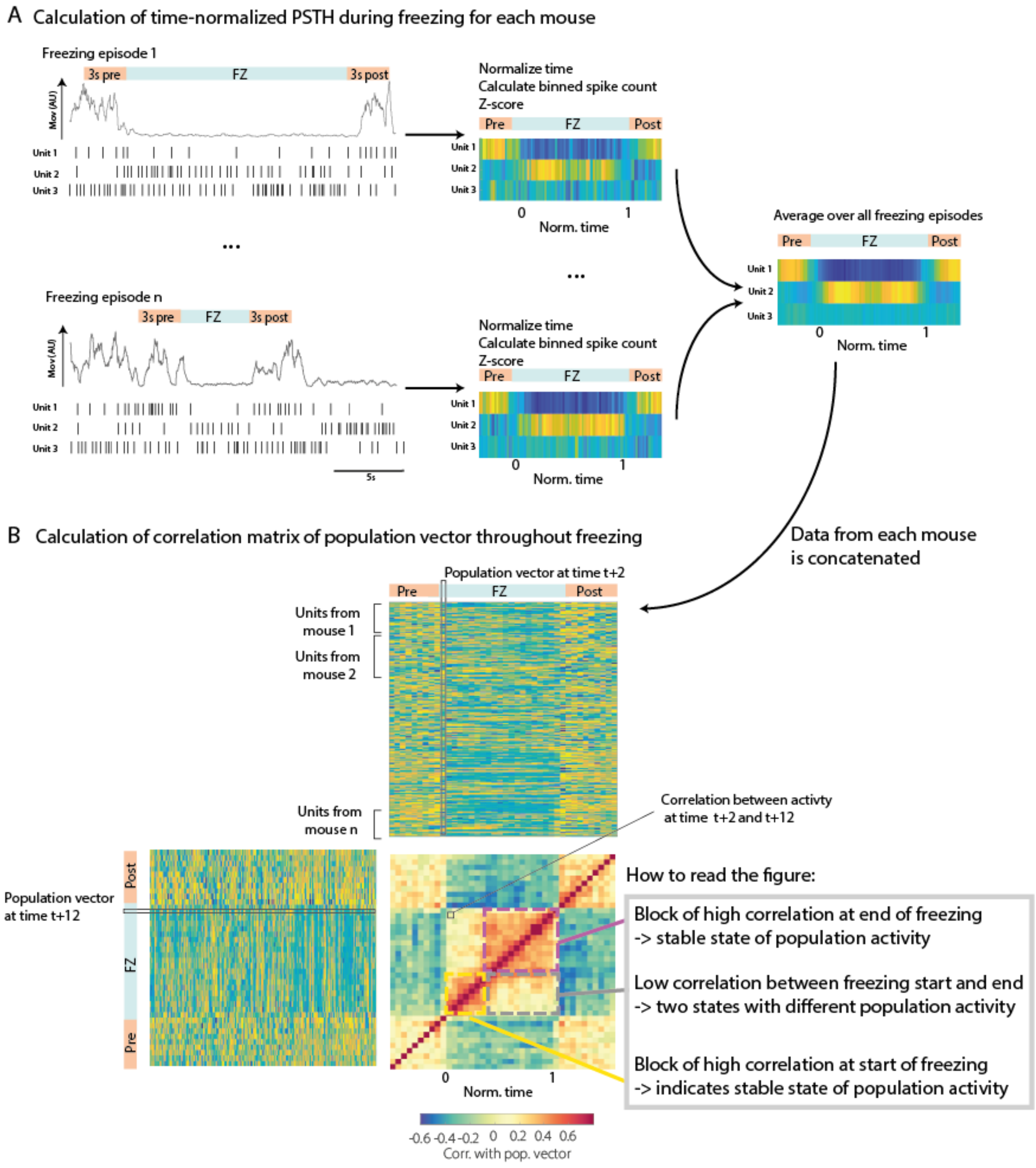

A. Schematic showing calculation for one mouse of PSTHs of 3 PFC units during a normalized freezing episode. Spiking activity from all freezing episodes was realigned in time between 0 (freezing start) and 1 (freezing end) in order to analyze the temporal evolution of population activity during freezing episodes regardless of length. Then this activity was binned to calculate a PSTH and z-scored. Finally activity from all freezing episodes was averaged.

B. Schematic showing calculation of the population vector correlation matrix. First, all unit PSTHs from all mice were grouped together in order to form a large pseudo-population that could be analyzed as a whole. The correlation matrix is produced by calculating for each pair of time bins the correlation between the population vectors at this time. At each position in the matrix, red indicates that the dmPFC population vectors are similar at the 2 different times and green indicates that they are different. Note that during freezing there is an initial phase of correlated activity that then transitions to a different type of activity which stabilizes until freezing end.
