# Supplementary material for: Breathing-driven prefrontal oscillations regulate maintenance of conditioned-fear evoked freezing independently of initiation
Source: Nat Commun. 2021 May 10;12:2605. doi: 10.1038/s41467-021-22798-6 (PMC8110519; doi:10.1038/s41467-021-22798-6)
Supplement: Supplementary file 3 — Reporting Summary [file 41467_2021_22798_MOESM3_ESM.pdf]

nature research

Corresponding author(s): Sophie Bagur

Last updated by author(s): 31/01/2021

Reporting Summary

Nature Research wishes to improve the reproducibility of the work that we publish. This form provides structure for consistency and transparency in reporting. For further information on Nature Research policies, see our Editorial Policies and the Editorial Policy Checklist.

Statistics

For all statistical analyses, confirm that the following items are present in the figure legend, table legend, main text, or Methods section.

n/a	Confirmed	
☐	☒	The exact sample size (n) for each experimental group/condition, given as a discrete number and unit of measurement
☐	☒	A statement on whether measurements were taken from distinct samples or whether the same sample was measured repeatedly
☐	☒	The statistical test(s) used AND whether they are one- or two-sided<br>*Only common tests should be described solely by name; describe more complex techniques in the Methods section.*
☒	☐	A description of all covariates tested
☐	☒	A description of any assumptions or corrections, such as tests of normality and adjustment for multiple comparisons
☐	☒	A full description of the statistical parameters including central tendency (e.g. means) or other basic estimates (e.g. regression coefficient) AND variation (e.g. standard deviation) or associated estimates of uncertainty (e.g. confidence intervals)
☐	☒	For null hypothesis testing, the test statistic (e.g. F, t, r) with confidence intervals, effect sizes, degrees of freedom and P value noted<br>*Give P values as exact values whenever suitable.*
☒	☐	For Bayesian analysis, information on the choice of priors and Markov chain Monte Carlo settings
☒	☐	For hierarchical and complex designs, identification of the appropriate level for tests and full reporting of outcomes
☒	☐	Estimates of effect sizes (e.g. Cohen's d, Pearson's r), indicating how they were calculated

Our web collection on statistics for biologists contains articles on many of the points above.

Software and code

Policy information about availability of computer code

Data collection	Electrophysiological recordings : RHD2000 interface software<br>Behavioural recordings : Custom-made Matlab programs
Data analysis	Spike soring : Semi-automated cluster cutting procedure using KlustaKwik and Klusters (http://neurosuite. sourceforge.net/)<br>Recordings pre-processing and visualisation : NeuroScope and NDManager (http://neurosuite.sourceforge.net/).<br>Two-way mixed repeated anova was performed using GraphPad Prism, GraphPad Software, La Jolla California USA.<br>All other analyses and statistics were performed with custom made Matlab (2018a) programs, based on generic code that can be downloaded at www.battaglia.nl/computing/ and http://fmatoolbox.sourceforge.net/.

For manuscripts utilizing custom algorithms or software that are central to the research but not yet described in published literature, software must be made available to editors and reviewers. We strongly encourage code deposition in a community repository (e.g. GitHub). See the Nature Research guidelines for submitting code & software for further information.

Data

Policy information about availability of data

All manuscripts must include a data availability statement. This statement should provide the following information, where applicable:

- Accession codes, unique identifiers, or web links for publicly available datasets
- A list of figures that have associated raw data
- A description of any restrictions on data availability

All data and code can be made available on request to the corresponding author.

Field-specific reporting

Please select the one below that is the best fit for your research. If you are not sure, read the appropriate sections before making your selection.

☒ Life sciences ☐ Behavioural & social sciences ☐ Ecological, evolutionary & environmental sciences

For a reference copy of the document with all sections, see nature.com/documents/nr-reporting-summary-flat.pdf

Life sciences study design

All studies must disclose on these points even when the disclosure is negative.

Sample size	Groups of 8-15 mice were used to constitute groups for behavioural comparison. These sample sizes are standard for the field. Given that optogenetic animals allowed comparison before and during stimulation (paired analysis) a smaller sample size was used (7) than for bulbecomty animals in which paired analysis was impossible.
Data exclusions	Prior to optogenetic experiments, electrophysiological response in OB to light stimulation was verified. CHR2 injected mice that did not show clear responses to stimulation were excluded. A posteriori histological verification showed this lack of response was linked to absent or very low viral expression likely due to failed injection of the virus during surgery.
Replication	Two types of replication can be found in the manuscript :<br>- Total vs partial bulbectomy gave the same results of reduction in freezing levels<br>- Optogenetics vs bulbectomy : both procedures gave the same result that reducing OB activity leads to reduced freezing levels
Randomization	Allocation to groups for behavioural tests (GFP/CHR2 or SHAM/bulbectomy) was random.
Blinding	Experimenters were not blind to the identity of the animals during testing since the experiment required recording and monitoring the quality of electrophysiological signals that revealed the animal's group : absence of OB recordings for bulbectomized individuals and response to laser stimulation in OB for CHR2 animals.<br>Animals were simultaneously tested in pairs, one from each group in two adjacent, separate chambers in the conditionning system and therefore were handled by the experimenter very close in time.<br>No subjective analysis was performed : all analysis are standardized computer anlaysis of signals established beforehand.

Reporting for specific materials, systems and methods

We require information from authors about some types of materials, experimental systems and methods used in many studies. Here, indicate whether each material, system or method listed is relevant to your study. If you are not sure if a list item applies to your research, read the appropriate section before selecting a response.

Materials & experimental systems

n/a	Involved in the study
☒	☐ Antibodies
☒	☐ Eukaryotic cell lines
☒	☐ Palaeontology and archaeology
☐	☒ Animals and other organisms
☒	☐ Human research participants
☒	☐ Clinical data
☒	☐ Dual use research of concern

Methods

n/a	Involved in the study
☒	☐ ChIP-seq
☒	☐ Flow cytometry
☒	☐ MRI-based neuroimaging

Animals and other organisms

Policy information about studies involving animals; ARRIVE guidelines recommended for reporting animal research

Laboratory animals	Mus musculus, C57Bl6, male, between 3 and 6 months old.<br>Mice were housed in an animal facility (08:00–20:00 light), one per cage after surgery.<br>Animal housing conditions were as follows : temperature:22°C, humidity : 45%, lunminance : 500 lux
Wild animals	No wild animals were used in the study
Field-collected samples	No samples were collected in the field.
Ethics oversight	All behavioural experiments were performed in accordance with the official European guidelines for the care and use of laboratory animals (86/609/EEC), in accordance with the Policies of the French Committee of Ethics (Decrees n° 87–848 and n° 2001–464) and after approval by ethical committee (reference : 2016-09). Animal housing facility of the laboratory where experiments were made is fully accredited by the French Direction of Veterinary Services (B-75-05- 24, 18 May 2010). Animal surgeries and experimentations

were authorized by the French Direction of Veterinary Services for K.B. (14-43).

Note that full information on the approval of the study protocol must also be provided in the manuscript.
